# Oncolytic virotherapy in cancer treatment: challenges and optimization prospects

**DOI:** 10.3389/fimmu.2023.1308890

**Published:** 2023-12-15

**Authors:** Lingjuan Chen, Mengsi Zuo, Qin Zhou, Yang Wang

**Affiliations:** ^1^ Cancer Center, Union Hospital, Tongji Medical College, Huazhong University of Science and Technology, Wuhan, China; ^2^ Institute of Radiation Oncology, Union Hospital, Tongji Medical College, Huazhong University of Science and Technology, Wuhan, China; ^3^ Hubei Key Laboratory of Precision Radiation Oncology, Wuhan, China; ^4^ Department of Oncology, The Sixth Hospital of Wuhan, Affiliated Hospital of Jianghan University, Wuhan, China; ^5^ National “111” Center for Cellular Regulation and Molecular Pharmaceutics, Key Laboratory of Fermentation Engineering (Ministry of Education), Cooperative Innovation Center of Industrial Fermentation (Ministry of Education & Hubei Province), College of Bioengineering, Hubei University of Technology, Wuhan, China

**Keywords:** oncolytic viruses (OVs), combination therapy, antitumor immunity, challenges, immune checkpoint inhibitor

## Abstract

Oncolytic viruses (OVs) are emerging cancer therapeutics that offer a multifaceted therapeutic platform for the benefits of replicating and lysing tumor cells, being engineered to express transgenes, modulating the tumor microenvironment (TME), and having a tolerable safety profile that does not overlap with other cancer therapeutics. The mechanism of OVs combined with other antitumor agents is based on immune-mediated attack resistance and might benefit patients who fail to achieve durable responses after immune checkpoint inhibitor (ICI) treatment. In this Review, we summarize data on the OV mechanism and limitations of monotherapy, which are currently in the process of combination partner development, especially with ICIs. We discuss some of the hurdles that have limited the preclinical and clinical development of OVs. We also describe the available data and provide guidance for optimizing OVs in clinical practice, as well as a summary of approved and promising novel OVs with clinical indications.

## Introduction

Oncolytic virotherapy is a promising emerging class of anticancer approaches (1). The foundation of oncolytic viruses (OVs) is that they infect and lyse tumor cells but leave normal cells intact (2). From the original natural virus to a gene-edited virus, the oncolytic virus has developed from herpes virus to more than ten commonly used viruses, including herpes simplex virus (HSV), adenovirus, vaccinia virus (VV), Newcastle disease virus (NDV), measles virus (MV), reovirus, coxsackie virus, poliovirus, and vesicular stomatitis virus (VSV). Oncolytic virus species span from single strand to double strand, from RNA to DNA, and from natural to gene editing, greatly improving the flexibility of this therapy in clinical treatment (3, 4).

OVs provide novel and promising treatment options for tumor patients resistant to traditional therapies. Currently, several viruses have already been approved, and some are being extensively investigated and are undergoing clinical trials in various types of advanced cancers. Despite the antitumor potential of OVs, there are still some unique limitations for developing OVs into a new class of anticancer drugs, including inadequate OV penetration and spread, host antiviral immunity, patient selection, and low or moderate efficacy when used as single agents. More studies are needed to overcome the preclinical and clinical challenges.

Therefore, in this review, we will discuss the mechanism and limitations of monotherapy. In particular, we will delve into the process of combination therapy, especially with ICIs. Ultimately, we will discuss the challenges and optimization strategies of oncolytic virotherapy to overcome these barriers.

## Mechanism of OV

### OVs directly lyse tumor cells

Normal host cells can clear viruses by activating signaling pathways. However, tumor cells lose antiviral ability and allow the replication of viruses (5, 6). OVs target tumor cells to selectively infect, replicate, and lyse cancer cells, leaving normal cells unharmed. OVs also release progeny virions that spread to uninfected cells, resulting in amplification of oncolytic activity (7, 8). OVs can be classified into naturally occurring viruses and genetically modified viruses, while most oncolytic viruses are not naturally but rather genetically modified for oncolytic activity. Naturally occurring OVs, such as reovirus, NDV, enteroviruses, and MV, were mainly used in their native forms. However, human pathogenic viruses such as HSV and adenovirus have been genetically modified (3). There are novel methods to genetically modify OVs instead of traditional time-consuming and labor-intensive methods. The Cerullo group described a new method called GAMER-Ad to genetically modify adenovirus genomes within 2 days, which consisted of using the well-described cloning method Gibson Assembly to replace the gp19k region with a gene of interest (9). The Zhang group also showed the “plug-and-display” decoration strategy by using SpyTag/Catcher synthesis for the design of virus-like particle (VLP)-based modular vaccines (10).

Several factors enhance OVs targeting tumor cells but sparing normal cells. This is due to aberrant signaling pathways in cancer cells that have effects on the loss of viral defense mechanisms, including the interferon (IFN), p53, and retinoblastoma (Rb) pathways and the induction of RAS/RAF/MEK/ERK pathways (11, 12).

First, viruses such as NDV, VSV, MV, and VV use the interferon (IFN)/protein kinase R (PKR) pathway for their natural oncotropism. In normal infected cells, IFN production activates the downstream Janus kinase signal transducer and activator of transcription (JAK/STAT) signaling pathway, leading to upregulation of PKR. The autophosphorylation of PKR and phosphorylation of the alpha subunit of eIF-2 inhibit protein synthesis and viral replication. However, most tumor cells deregulate the IFN pathway; thus, PKR remains unphosphorylated, and viral protein synthesis and replication continue (13, 14).

Additionally, the hyperactive RAS signaling pathway in tumor cells is another common feature leading to inhibition of PKR and allowing replication of OVs such as reovirus, HSV, and VV (15).

On the other hand, cancer cells that harbor the retinoblastoma (Rb) pathway and deregulated E2F activity will enable OVs such as adenovirus, reovirus, VV, and HSV to replicate (16). Viruses such as adenovirus and reovirus prefer to replicate in p53-deficient cancer cells (17). Adenovirus enters the cell cycle of host cells via E1A and E1B expression for replication, while E1A interacts with Rb and blocks the E2F transcription factor, allowing viral replication. Adenovirus prefers to replicate in Rb-deficient cells, and a 24-base pair deletion in E1A makes them conditionally replicate in tumor cells while leaving healthy cells intact (18).

### OVs induce antitumor immunity

OVs stimulate innate immune responses. After administration, viral elements, known as pathogen-associated molecular patterns (PAMPs), including proteins, DNAs, RNAs and viral capsids, are exposed to the host immune system (13), which induces immunogenic cell death (ICD). ICD is the basis for OVs to elicit antitumor immunity, which includes not only immunogenic apoptosis but also necroptosis, necrosis, autophagic cell death, and pyroptosis of cancer cells (19–21).

Furthermore, immunogenic cell death (ICD) caused by OV exposure leads to the release of tumor-associated antigens (TAAs), tumor-associated neoantigens (TANs), and damage-associated molecular pattern molecules (DAMPs) (22, 23). PAMPs/DAMPs then trigger the overexpression of cytokines and chemokines such as type I interferons (IFNs), TNF-a, IL-6, IL-1, CCL2, CCL3, CCL5, and CXCL10 (24). Chemokines and cytokines take part in recruiting neutrophils and macrophages infiltrating infection sites as well as stimulating the activity of NK cells and DCs, which further activates the innate response and turns immunologically “cold” tumors into “hot” tumors (25).

OVs also induce adaptive immunity against tumor cells mainly through the tumor-specific T-cell response. After tumor cells are infected by OVs, type I IFNs activate MHC class I and II molecules (26) and costimulatory molecules on the surface of DCs, including CD40, CD80, and CD86 (27–29), which contribute to APC maturation for recruiting and reactivating T cells. Activated CD8+ T cells and B cells clear newly grafted tumors as well as distant tumors in an OV-independent manner (30, 31). Many OVs can generate specific T-cell immunity in an antigen-specific manner against cancer, including HSV-1 (32), oncolytic VV (OVV) (33) vesicular stomatitis virus (VSV) (34), MeV (35), and OAd (36).

### OVs affect the tumor extracellular matrix

The extracellular matrix (ECM) is formed by activated cancer-associated fibroblasts (CAFs) and constitutes a solid tumor mass (37). The rigid ECM made by excessive accumulation of collagenous matrix, proteoglycans, and hyaluronan forms a physical barrier that hundles OVs to effectively arrive at the whole tumor mass (38). At the same time, tumor cells can produce high levels of fibroblast growth factor 2 (FGF2) to render them sensitive to viral infection (39). Many gene-editing OVs are focused on enhancing crosstalk between CAFs and cancer cells (39, 40), such as OAd targeting glioblastoma−associated stromal FAP+ cells as well as glioblastoma cells (40).

### OVs affect tumor vasculature

OVs are capable of infecting and lysing vascular endothelial cells (VECs) to affect tumor vasculature. OV HSV-1716 was first reported to exert direct antiangiogenic effects and contribute to the overall therapeutic efficacy in ovarian carcinoma (41). Vascular endothelial growth factor (VEGF) enhances the sensitivity of tumor vessels to VV infection, depresses the antiviral response by Erk1/2 and Stat3 signaling and upregulates the expression of PRD1-BF1/Blimp1 in the tumor vasculature (42). Engineered OVs can selectively target and disrupt the tumor vasculature (43). VSV was shown in a murine colon cancermodel replicating in the tumor neovasculature as well as spreading throughout the tumor mass through three-dimensional imaging (44).

## Limitations of monotherapy and advances in combinatorial therapy

### Limitations of monotherapy

Although many tumor-selective mechanisms were validated for OVs preclinically (45, 46) and clinically (47–50), their efficacy was limited when administered as monotherapies (2). There are several potential reasons for the modest activity of systemic OV monotherapy. First, neutralizing antiviral antibodies induced by treatment or preexisting antibodies may hinder OVs from replicating in and lysing tumors (51, 52). Second, antiviral resistance mechanisms, which include complement activation, antiviral cytokines, and macrophages, might promote the rapid clearance of OVs (53, 54). These antiviral immunities may present a major hurdle for OVs, although the effects of antiviral immunity are not well defined, and intratumoral OV therapy might overcome this problem with local and abscopal effects. Interestingly, while preexisting immunity to NDV limits its replication in tumors, tumor clearance, abscopal antitumor immune effects, and survival are not compromised but are superior in NDV-immunized mice with repeated therapeutic dosing. These studies provide a clinical rationale for repeated dosing therapy (51). Third, the extracellular matrix, fibrosis, necrosis, and interstitial hydrostatic pressure may act as an insuperable physical barrier to prevent OVs from entering cellular receptors expressed in tight junctions, which has attracted the attention of many scholars to overcome this challenge (52, 55, 56). Fourth, transgene expression or engineering for tumor selectivity may cause loss of viral fitness and reduce replication competence and oncolytic activity (57, 58). Fifth, the expression of transgenes may induce clearance of OVs from the substantial immune response, which promotes continuous optimization and updates of transgenes (59). Based on the lack of durable response with monotherapy, research efforts have largely focused on selecting both a virus and a combination partner.

### The basis of combination therapy

**Figure 1:**
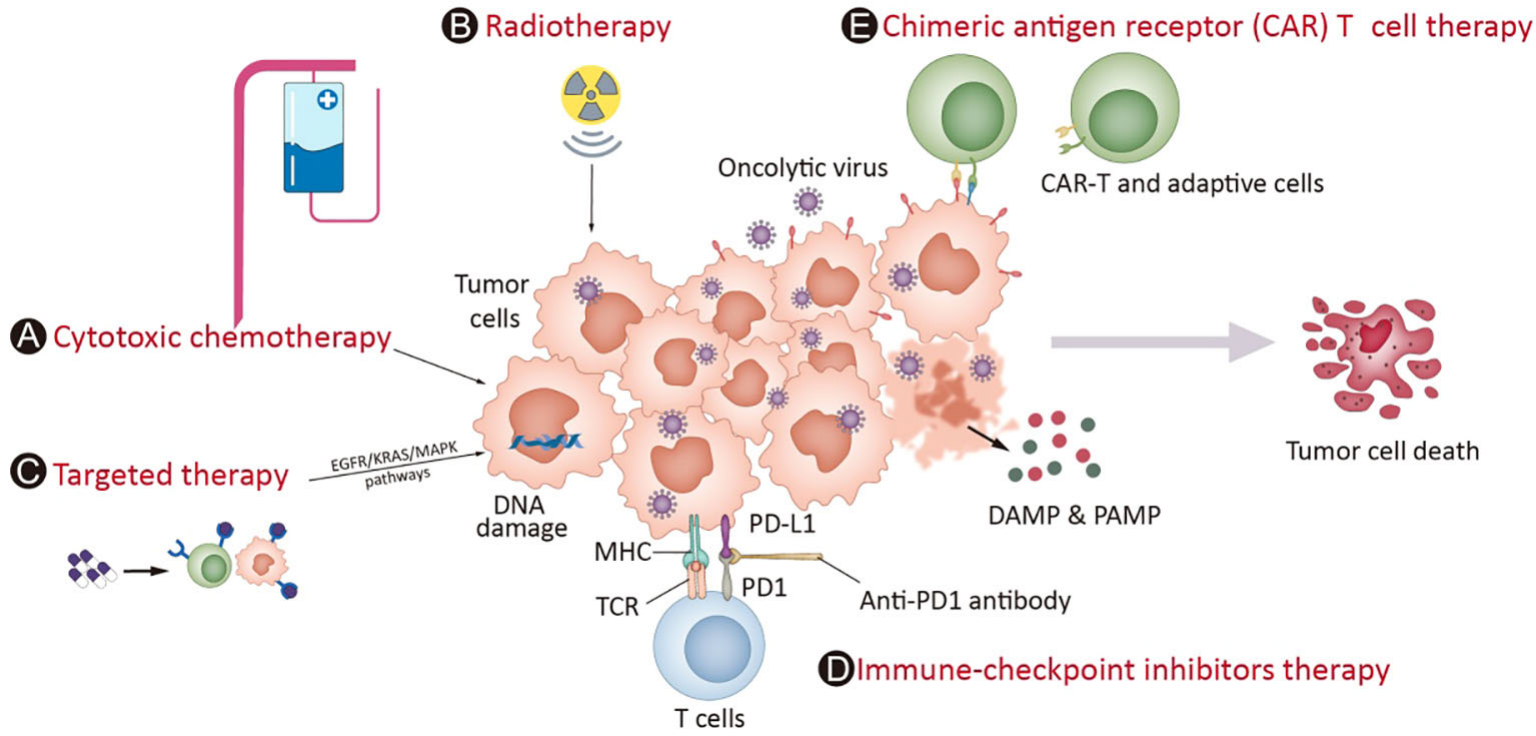
OV potential combination strategies in the clinic. Mostoncolytic viruses (OVs) directly lyse tumor cells, increasing the release of DAMPs, PAMPs and cytokines and promoting the accumulation of CTLs in tumor beds and retention of their killing capability. Cytotoxic chemotherapy **(A)** and radiotherapy **(B)** complement virotherapy through various mechanisms, such as hindering antiviral immune responses, improving tumor cell immunogenicity via ICD and directly killing cancer cells. Targeted therapies **(C)** often interrupt abnormal signaling pathways and cause tumor cell death, which induces weak or moderate immune responses. The infection of OVs leads to overexpression of immune checkpoint molecules such as PD-L1 and CTLA-4 from cancer cells and promotes a “hotter” immune microenvironment, which provides synergistic effects on the combination of ICIs **(D)**. Chimeric antigen receptor (CAR) T cell therapy **(E)** involves T cells expressing genetically modified CARs, which enables them to kill tumor cells with corresponding specific antigens. The combination of CAR-T-cell therapy with genetically modified OVs can greatly attract CAR-T-cell penetration into the tumor mass through the overexpression of cytokines in the tumor microenvironment and enhance the treatment effect of CAR-T cells in cancers.

Given this, combinatorial therapy with OVs has become an attractive option. Furthermore, the mechanisms of OVs are distinctly different from those of other anticancer therapies, and the toxicity profiles generally do not overlap with those of other treatments (60, 61); at the same time, OVs can be administered repeatedly if needed. These features make OVs a rational candidate for inducing personalized immune responses and combining them with most other treatment modalities, including chemotherapies, radiotherapy, targeted therapies, and immunotherapies such as immune checkpoint inhibitors (ICIs), chimeric antigen receptor (CAR) T-cell and adoptive T-cell therapies. When deciding the relative merits of combining the OV with another agent, several factors must be considered, such as understanding the intrinsic lytic as well as immune-modulatory properties of the virus, and factors including the site of action, duration of therapy needed and cost of goods should also be considered (62). The synergistic effects have been tested for OVs combined with chemotherapy or radiotherapy in many studies (3, 63, 64). One recent review (65) summarized the key molecules in relevant signaling pathways, such as EGFR-KRAS (e.g., KRASG12C), PI3K-AKT-mTOR, ERK-MEK, JAK-STAT, p53, PD-1-PD-L1, and epigenetic or immune pathways (e.g., histone deacetylases, cGAS-STING), which are currently under investigation and have the potential to be combined with OV. On the other hand, the induction of a systemic immune response by OVs to turn ‘cold’ tumors into ‘hot’ tumors could increase susceptibility to immunotherapy approaches such as ICIs (66). The combination of CAR-T-cell therapy with genetically modified OVs can significantly induce CAR-T cells to recognize and penetrate tumors (67). These combinations might be effective in overcoming the flaws of each component to further enhance the outcome (**Figure 1**).

### Combination of OVs and immune checkpoint inhibitors

The most advanced combination regimens in the clinic are those with ICIs, with initial data suggesting promise (68). Advances in the development of ICIs have changed the frame of current cancer treatment. OV infection can lead to the upregulation of immune cells as well as immune checkpoint molecules in the TME. Liu et al. armed OVV with IL-2 to effectively modify the cancer-immune set point, and combination with an anti-PD-1/PD-L1 antibody could cure most late-stage tumors in mice (69). Bo et al. revealed that oncolytic HSV2 upregulates PD-L1 expression in the TME (70). In addition to immune checkpoint molecules expressed on T cells and tumor cells, OVs have also been shown to upregulate immune checkpoint molecules primarily located on NK cells. Wang et al. reported that oncolytic HSV2 upregulates NKG2A expression on NK cells and that anti-NKG2A antibodies enhance the antitumor effects of UV light-inactivated oHSV2-stimulated NK92 cells *in vitro* and *in vivo* (71). Nakao et al. reported that intratumoral expression of IL-7 and IL-12 using an oncolytic virus increases systemic sensitivity to immune checkpoint blockade (72). The upregulation of immune checkpoint molecule expression can provide targets for subsequent combination therapy with immune checkpoint inhibitors in clinical studies. Large-scale phase III trials have established the role of oncolytic viruses, which not only lyse cells to obtain cancer-killing effects but also cause attractive alterations in the tumor immune microenvironment. “Priming” by viral infection can change the ‘cold’ TME into ‘hot’ with a multitude of immune cells and cytokines, which directs the phase for subsequent ICI delivery. The number of clinical trials studying combinations of OVs and ICIs continues to rise, and some with initial data from these trials have suggested promising therapeutic potentials with good safety profiles (68).

T-VEC is leading this promising combination immunotherapy. The phase Ib stage of MASTERKEY-265 showed promising tumor responses with the combination of T-VEC plus pembrolizumab (73). Another phase 1b clinical trial also showed a confirmed objective response rate of 62%, with a complete response rate of 33% with the combination of T-VEC and pembrolizumab (74), which suggests the impact of T-VEC on cytotoxic T-cell infiltration as well as enhancing the efficacy of ICI therapy by modifying the TME (74). Sun et al. reviewed a case series and showed an overall response rate of 90% when T-VEC was combined with ICIs (including pembrolizumab, ipilimumab/nivolumab, or nivolumab) for unresectable stage III IV melanoma treatment, which suggested that the combination could provide synergistic effects for patients (75). ONCOS-102, an oncolytic Ad5 armed with GM-CSF, has also shown promising antitumor effects in combination with pembrolizumab in advanced or unresectable melanoma (NCT03003676). Some ongoing clinical trials combining OVs with ICIs are shown in **Table 1**. This therapy has great potential to improve cancer treatments in the near future, with growing numbers of OVs and ICIs entering clinical development.

**Table 1:** Clinical trials of OV combined with ICIs.

ClinicalTrials. gov identifier	OV	Checkpoint inhibitor	Indication	N	Response data
NCT01740297	T- VEC	Ipilimumab	Melanoma	198	ORR 39% (T- VEC + ipi) versus 18% (ipi); P = 0.002
NCT02263508	T- VEC	Pembrolizumab	Stage IIIB–IV melanoma	21	48% ORR
NCT03153085	HF10	Ipilimumab	Melanoma	28	N/A
NCT02272855	HF10	Ipilimumab	Melanoma	46	BORR at 24 weeks 41%; median PFS 19 months; median OS 21.8 months
NCT02565992	CAVATAK	Pembrolizumab	Melanoma	50^a^	N/A
NCT03003676	ONCOS-102	Pembrolizumab	Advanced or unresectable melanoma	12^a^	N/A
NCT02626000	T- VEC	Pembrolizumab	HNSCC	36	ORR 16.7% and disease control rate 38.9%
NCT02636036	Enadenotucirev	Nivolumab	Metastatic or advanced- stage epithelial tumors (CRC, bladder, HNSCC, salivary gland cancer)	30^a^	N/A
NCT02509507	T- VEC	Pembrolizumab	HCC,liver metastases	244^a^	N/A
NCT03071094	Pexa- Vec	Nivolumab	First- line HCC	30^a^	N/A
NCT03647163	VSV- IFNβ-NIS	Pembrolizumab	NSCLC and HCC	23^a^	N/A
NCT02879760	MG1-MAGEA3 + Ad MAGEA3	Pembrolizumab	NSCLC	61	N/A
NCT02043665	CAVATAK	Pembrolizumab	NSCLC and bladder cancer	90	N/A

T- VEC, talimogenelaherparepvec; VSV- IFNβ-NIS, vesicular stomatitis virus encoding the interferon-β transgene and sodium–iodide symporter; MG1-MAGEA3, Maraba virus expressing melanoma-associated antigen A3; Ad MAGEA3, adenovirus vaccine expressing melanoma-associated antigen A3; HNSCC, head and neck squamous cell carcinoma; CRC, colorectal cancer; HCC, hepatocellular carcinoma; NSCLC, non-small cell lung cancer; ORR, objective response rate; ipi, ipilimumab; BORR, best objective response rate; OS, overall survival; N/A, not available; PFS, progression-free survival; a Estimated enrollment.

There are still some noteworthy issues in the combination of OVs with ICIs, such as different dosing sequences. This includes alternate administration modes (NCT05228119, NCT05644509), sequential administration (73) (NCT02798406), and simultaneous administration of OVs and ICIs [NCT02263508 (74), NCT03647163 (76)]. Based on the characteristics of OVs and immune checkpoint inhibitors, different dosing sequences may significantly impact the results of clinical trials.

The influence of the dosing scheme is also evidenced by clinical studies involving T-VEC and PD-1 inhibitors in melaloma. Though the phase Ib stage of MASTERKEY-265 showed promising tumor responses with the combination of T-VEC plus pembrolizumab (77). The later phase III trial of MASTERKEY-265 combining T-VEC with pembrolizumab did not yield significant improvements in PFS or OS compared to the placebo-pembrolizumab combination (74), which might be due to different dosing schemes and the subsequent influence on the TME. Notably, the combination therapy strategies in phase 1b and phase 3 trials exhibited discrepancies, encompassing variations in the timing of the initial administration of pembrolizumab in conjunction with OVs and the intervals between each combination therapy session.

## Challenges of oncolytic virotherapy

### Preclinical challenges

#### Mouse models

There are several limitations that might influence the evaluation of OVs with currently available mouse models. First, the highly restrictive cell tropism of many mammalian viruses that are designed to be applied to humans hinders their activation in mouse models. Vaccinia virus is generally tropic for most cell lines, including mouse- and human-derived cancer, whereas most mouse cells are resistant to HSV1, with a few notable exceptions, including A20 lymphoma and D4M melanoma cell lines (32). Productive infection of human adenoviruses in murine cells is significantly lower than in human cells (78), although infection and some replication can be seen (79). That is why most work on adenovirus immunology has utilized replication-defective vaccine vectors, and knowledge on the role of immune responses to replicating adenoviruses is very limited (80). The Syrian hamster model is both immunocompetent and replication-permissive, with which human adenovirus replicates well and confirmed replication, and has become a valuable tool for the field of oncolytic adenovirus (81). However, in most accasions, immunocompromised mouse strains, immunocompetent hosts with intact adaptive immune systems, or even humanized mouse models might be needed under certain circumstances. However, they might lack specific cell types or molecules for understanding the interaction of OVs with the human immune system (32). In addition, subcutaneous tumor models cannot reflect the disease features of multiple metastatic lesions and do not provide an accurate status of the TME. Orthotopic mouse models are available to provide a comparable microenvironment, but leading the virus into the tumor site is more challenging. Patient-derived xenograft mouse models often have long latency periods, although they can provide a genetic landscape more accurately (82). Patient-derived organoid (PDO) models, which maintain the heterogeneity, hypoxic microenvironments, and diversity of differentiated states seen in primary tumors, can serve as viable tools for testing OVs (83, 84). The response of oncolytic adenovirus in renal cell carcinoma patient-derived organoids (85) and pancreatic patient-derived organoids (PDOs) (86) indicates that PDO sensitivity to OVs could be informative.

#### Balance between antiviral and antitumor immune responses

The balance between antiviral and antitumor immune responses to achieve optimal tumor regression is another challenge for OVs (87, 88). Especially for HSVs or adenoviruses derived from endemic viruses, cross-reactive antibodies might exist and impair effective viral replication when previously exposed to viruses of the same family (89, 90). Furthermore, advanced-stage tumors might require repeated OV injections, which might promote the appearance of neutralizing antibodies. Several strategies are attempting to reduce antiviral immunity. Some approaches using protective coatings with chemical polymers, liposomes, or cell-derived nanovesicles to physically protect OVs against immune factors are being investigated (91–93), although these protectively coated OVs are difficult to store and have higher manufacturing costs.

Ex vivo OV-loaded cells are one strategy for limiting early viral clearance. Tumor-infiltrating immune cells, including mesenchymal stem cells, macrophages, dendritic cells, and T cells, can infiltrate tumor sites and are available as cellular carriers for OVs (94, 95). Delivering OVs to the brain through certain cellular carriers is a good method for treating tumors of the central nervous system. For example, adeno virus-based OV ICOVIR17 carried by tumor-tropic mesenchymal stem cells demonstrated extended survival in glioblastoma models as well as amassing of OV in brain lesions (96).

#### Biomarkers

Furthermore, investigating potential predictive biomarkers for OV therapy is another challenge and is at a very early stage of development. There are several directions for biomarkers to assess the efficacy of OVs. Using viral DNA or protein expression as biomarkers or using imaging can assess whether OVs have reached the target tumor. Alternatively, lysis can be evaluated to assess tumor killing or tumor shrinkage through imaging techniques. Furthermore, by detecting evidence of a transition from immunologically cold to hot, biomarkers can assess whether OV modulates the TME.

Some potential biomarkers to assess the pharmacodynamic activity of OVs are also under way, which include assessing specific gene expression in cancer or assessing specific viral gene expression (97)(29164063). Cathepsins B and L are useful biomarkers for the efficacy of reovirus-mediated tumor cell killing (98), and serum high-mobility group box 1 (HMGB1) protein was reported to be a potential predictive and prognostic biomarker for adenoviruses combined with immunotherapy (99).

Studies in preclinical models suggest that JAK deficiency might be associated with the antiviral immune response and viral replication in HSV1 and VSV (100). Studies of melanoma models show that STING deficiency improves OV replication and lysis by T-VEC (101). One example of an imaging technique to track a virus noninvasively was a measles virus engineered to express the human thyroid NIS (102).

Other potential molecular and/or cellular characteristics for predicting clinical benefit are under consideration, including systemic characteristics, immune-related characteristics, and tumor-intrinsic characteristics (103). Systemic features include circulating tumor DNA and exosomes, mainly in the peripheral circulation. Immune-related features refer to the activation of innate immunity as well as the induction of infiltrating tumor-reactive lymphocytes. Tumor-intrinsic characteristics include tumor mutational burden, tumor cell PD-L1 expression, inflammatory/antiviral gene expression, and factors related to the TME. Further research is necessary to clarify potential biomarkers, which should be acknowledged in future clinical trials (103).

### Clinical challenges

#### Delivery challenge

Intratumoral administration of oncolytic viruses remains the predominant delivery method to date. In preclinical and clinical trials, following intratumoral administration of oncolytic viruses, a reduction in both injected and noninjected tumor sites suggests that intratumoral viral delivery exhibits distant effects. The conflict between the clinical demand for intravenous delivery and the need for intratumoral injection is the major challenge to the clinical application of OVs. Intravenous delivery enables widespread OV infection to all lesions and avoids the need for localization technicians, especially when tumors are physically inaccessible. Intravenous delivery offers many advantages, but several drawbacks should also be considered. First, viral particles could be precociously cleared by neutralizing antibodies, further limiting the effect. In addition, the optimal dose is undetermined after the virus is diluted in peripheral circulation, which makes bioavailable titers at the tumor site unpredictable. Thus, innovative strategies for evading neutralization must be developed. Various approaches, including retargeting (104, 105), the use of cell carriers (106, 107), coating with polymers (108, 109), and encapsulation in liposomes (110, 111), have been explored to shield oncolytic viruses from neutralizing antibodies.

Some early-phase clinical trials are investigating the intravenous delivery method of OVs. One study of enadenotucirev, a chimeric oncolytic adenovirus, confirmed that viral particles were consistently detected on resected tumors after the virus was administered intravenously before surgery (112). Another study of the oncolytic vaccinia virus Pexa-Vec, which was given intravenously to melanoma and colorectal cancer patients prior to surgery, showed a tolerable safety profile and confirmed the existence of OVs in resected tumor specimens (113). Recent advancements in nanotechnology and its application in delivering nucleic acids are paving the way for novel carrier systems to overcome the challenges associated with intravenous (IV) administration of oncolytic viruses. Kennedy et al. pioneered a nanoparticle-based delivery platform capable of facilitating repeated IV administration of viral immunotherapies (114).

#### Safety concerns

OVs are vigorously replicating viruses and need attention related to the risks of unconscious transmission from patients to other contacts and the environment when used in the clinic. Guidelines and protocols for storage, handling, and administration, processes to operate accidental spills and overdoses, and suitable sterilization of contact areas are all needed, and several guidance protocols are now available (115–117). To date, no reported transmission to contacts or exposures has been presented. One study showed that 8.4% of household exposures with T-VEC reported cold sores, who were not confirmed to have infections and had clinically mild symptoms (112). Concerns also exist regarding viral shedding, especially in immunosuppressed patients, and the potential of OVs containing recombinant DNA elements to recombine with naturally occurring wild-type viruses. In a study of 97 clinical trials (118), no reported transmission of OVs to contacts or exposures was presented. The presence of viruses in tissue was studied to understand the biodelivery of viruses to tumor sites and to search for tissues and/or fluids that may be reservoirs or sites of viral shedding. The most common site evaluated for OV shedding was blood or serum, followed by urinary shedding and tumor biopsy specimens. Salivary fluid, oral swabs, and other fluids or tissues, including cerebrospinal fluid, peritoneal washings, and injection sites, have also reported viral shedding.

## Optimizing oncolytic virotherapy

### Arming strategies

A dramatic feature of OVs is that they can express transgenes through genetic modification, which further increases their functionality. Backbone virus properties of intrinsic lytic and immune-modulatory features, the site of virus action, duration of therapy, and cost of goods should be taken into consideration when gene modification occurs (62).

### OVs armed with antigens

OVs can generate vaccine-like responses through the expression of a TAA in cold tumors, such as oncolytic vaccinia virus expressing ERBB2 (119) or Maraba MG1 rhabdo virus encoding melanoma-associated antigen 3 (MAGEA3) (120). Furthermore, OV replication and spread can induce T cells (121, 122), and this property could be augmented by transgenes involved in the homing of T cells (such as the expression of chemokines such as CCL19) (123). Armed OVs could express specific antigens on infected tumor cells and be specifically recognized by CAR-T cells, which enabled OVs to be a good combination with chimeric antigen receptor (CAR)-T therapy (124–127).

OVs armed with bispecific (CD3 and TAA) T-cell engager (BiTE) molecules could overcome the shortcoming of BiTE, which has a short half-life in serum, but OV replication could prolong the expression of BiTE. This strategy was first reported in VV, which encodes a secretory bispecific T-cell engager consisting of two single-chain variable fragments specific for CD3 and the tumor cell surface antigen EphA2 (EphA2-T-cell engager-armed VV (EphA2-TEA-VV)), and showed potent antitumor activity in comparison with control VV plus T cells in a lung cancer xenograft model (128). OHSV2 armed with BsAb molecules targeting PD-L1/CD3 could enhance T-cell-mediated tumor lysis *in vitro* regardless of PD-L1 high/low expression on tumor cells (129). OVs armed with BiTEs have shown activation of cytotoxic T cells as well as oncolysis to produce immune-mediated destruction of tumors not only in primary ex vivo patients but also in *in vivo* xenograft models (130, 131).

### OVs armed with Th1-stimulating cytokines

Armed OVs expressing Th1 cytokines could activate T-cell migration, proliferation, and homing to the TME and enhance the antineoplastic immune response (132), as well as successfully combine with CAR-T-cell therapies in xenograft tumor models (133). Cytokines, such as GM-CSF, IL-2, IL-12, and IFN-α, play very important roles in cancer treatment, but cytokines generally have short half-lives, act over short distances, and need to be repeatedly administered in short intervals to maintain efficient bioavailability, which limits their widespread clinical use. Scientists are making efforts to ensure that cytokines are locally expressed in tumors and to thus enhance OV antitumor activity as well as control side effects. Using OVV as an example, Liu et al. generated several membrane-bound vaccinia virus-armed cytokines, such as IL-2 (69), IL-12 (134), or IL-23 (135), to avoid potential systemic toxicity and can also induce potent antitumor effects, especially when combined with an anti-PD-1/PD-L1 antibody, by curing most late-stage tumors in mice. Other Th1-cytokine-armed OVVs, such as IL-7, IL-12 (72), IL-10 (136), IL-15 (137), IL-21 (138), IL-24 (139), and IL-36γ (140), have also been developed and shown to be effective and safe in a variety of tumor models.

### OVs armed with ICBs to alleviate immune suppression

Armed OVs with ICBs engineered to express checkpoint inhibitor antibodies with viruses to block PD-L1 or CTLA-4 could obtain even better antitumor activity than OVs combined with ICB therapy. Various studies have demonstrated the benefits of these armed OVs expressing ICB molecules (141–144).

These schemes also have limitations. First, armed OVs express tumor-localized ICB, while the maximum benefit of ICB requires immune cells in the periphery but not only within the tumor. In addition, the immune response might require ICB and OV on different schedules.

One study of measles viruses engineered to express anti-CTLA-4 showed a better effect in controlling tumor growth, while the survival effects were similar between viruses expressing anti-CTLA-4 or anti-PD-L1 antibodies and the control group (143). Variability might be due to different antitumor immunity cycles for CTLA-4 in the generation stage of T-cell responses but PD-L1 in the effector phase.

Arming strategies to enhance the effects of ICBs include molecules that influence the early stage of immune responses. One study showed that a Newcastle disease virus armed to express inducible T-cell costimulator (ICOS) ligand, which is necessary for the survival and function of T cells (145), not only induced T-cell infiltration in tumors but also enhanced antitumor efficacy when combined with a CTLA-4 blocking antibody (146). One other agent, OX40 or its ligand (OX40L), a costimulatory molecule that activates T cells (147), has been explored in combination with OVs (148, 149), but an increased T-cell response was not transferred to corresponding tumor inhibition when an OX40 agonist was combined with VSV-IFNβ (149). These studies suggested that research on armed OVs needs to identify the stage and schedule, not only combination strategies.

### Overcoming *in vivo* barriers to response

Substantial barriers, including physical and immunological factors, limit the clinical benefits of OVs. Approaches other than arming and combination factors should be undertaken to overcome *in vivo* barriers and enhance treatment responses.

The TME, similar to cancer-associated fibroblasts, can cause the deposition of extracellular matrix, which hinders the distribution of OVs, results in small loci of replication, and restricts immune cell migration into cancers (150). Armed OVs with transgenes to change the extracellular matrix components, including hyaluronidase, decorin, or relaxin, by degrading the ECM might improve viral spread, normalize the vasculature, and induce immune cell infiltration (151–153).

Poor penetrance for mislocalization can negatively influence the delivery of OVs. Inhalers for aerosol delivery offer a noninvasive method for delivery to the lung (154). OV delivery with ultrasound cavitation, a technique using ultrasound with microbubble formulation, can increase replication and spread (155), as well as intratumoral uptake of systemically delivered vaccinia virus (156). The application of cationic lipids (157) or pegylation (PEG) (158) might weaken the liver sequestration and toxicity of OVs.

In most accasions, anti-viral immunity presents a major hurdle for systemically administered OVs, and studies are trying to reduce vector neutralization and promote the T-cell response to overcome immunological barriers. Strategies to evade this, including cell carrier tropism for tumor tissues, such as peripheral blood mononuclear cells, which act as carriers for oncolytic reoviruses (159), can enable OVs to be shielded intracellularly. Moreover, antibody-blinded viruses, where nAb epitopes on viruses have been preidentified and mutated, can overcome the influence of OV delivery even with preexisting immunity (160). On the other hand, the effects of anti-viral immunity can also be a double-edged sword. Zamarin’s group demonstrated that preexisting immunity to NDV may increase its therapeutic efficacy through the potentiation of systemic antitumor immunity (51). Another study also showed that the anticancer efficacy of an HSV-1 OV can be enhanced by preimmunization and multicycle administration (161). The Cerullo‘ group also showed that preexisting antiviral immunity might enhance OV-induced antitumor immunity in oncolytic adenovirus (162).

Poor T-cell priming can lead to a lack of viral recognition and a lack of lysis. Homologous (repeated doses of the same virus) and heterologous (multiple doses of different viruses) prime-boost regimens can enrich the T-cell response and promote priming by OVs. One study reported in 2017 showed that the combination of three different treatments (priming with systemically delivered Reovirus, followed by double boosting with systemic VSV-ASMEL and anti-PD-1) significantly enhanced survival, with long-term cures, compared to any individual, or double, combination therapies, associated with strong Th1 and Th17 responses to tumor antigens, which indicated that it is possible to generate fully systemic, highly effective antitumor immunovirotherapy by combining oncolytic virus therapy (163).

## Approved and promising novel OVs in different cancers

Numerous OVs are currently in clinical progress. Different tumors, including melanoma, liver cancer, head and neck cancer, glioma, bladder cancer, pancreatic cancer, nasopharyngeal cancer, and lung cancer, are treated by OVs registered for clinical trials. One review (118) included 97 studies reporting data in 119 papers from 2000 to 2020 regarding OVs and showed that adenovirus is the most popular OV in clinical trials and that the most common tumor studied was melanoma. Here, we describe several OVs already approved (**Table 2**) and those in the later phase of clinical progress with hopeful clinical indications.

**Table 2:** Currently approved viruses worldwide.

Name	Virus	Indication	Location	Results from registry studies
Oncorine(H101)	Adenovirus Serotype 5	In combination with chemotherapy for patients with NPC	China (2005)	ORR 72.7% versus 40.3%
Imlygic(T-VEC)	HSV1	Unresectable stage IIIB–IV melanoma	Australia(2016), Europe(2015),Israel (2017), USA(2015)	DRR 16.3% versus 2.1%; median OS 23.3 months versus 18.9 months, HR 0.79, (P = 0.051)
Delytact (Teserpaturev)	HSV1	R/R glioblastoma following radiotherapy and temozolomide	Japan (2021)	Median PFS 4.7 months; median OS 20.2 months;
Rigvir(ECHO-7)	Echovirus	Stage I–II melanoma	Armenia (2016), Georgia (2015), Latvia (2004)	Decreased risk of disease progression with ECHO-7 relative to other experimental immunotherapies, HR 6.67 (P < 0.001)

T-VEC, talimogenelaherparepvec; HSV1, herpes simplex virus type 1; BCG, bacillus Calmette–Guerin; DRR, durable response rate; ORR, objective response rate; OS, overall survival; PFS, progression-free survival.

### Melanoma

Melanoma is a good candidate for the treatment of OV. Talimogenelaherparepvec (T-VEC), an engineered oncolytic herpes simplex virus type 1 (HSV1), was evaluated in a prospective randomized trial in patients with accessible and unresectable melanomas. The durable response rate (DRR) was 16.3% in patients receiving T-VEC versus 2.1% in those receiving GM-CSF, as well as improvements in overall survival (OS) (23.3 months versus 18.9 months), which led to full FDA approval in 2015 (48). T-VEC has since also been approved for use in Europe, Australia, and Israel.

ECHO-7, a genetically nonmodified, oncotropic and oncolytic echovirus, was first approved in 2004 in Latvia and then in Georgia and Armenia for the decreased risk of disease progression relative to other experimental immunotherapies, HR 6.67 (P < 0.001) (164).

There are still some ongoing trials for the therapy of melanoma with OVs. HF10, an HSV1-based OV, is being assessed with metastatic or unresectable melanomas combined with ipilimumab (165) or as monotherapy in advanced-stage cutaneous solid tumor patients (166). Preliminary reports showed that the ORR at 24 weeks was 41%, 68% of patients had stable disease, the median PFS was 19 months, and the median OS was 21.8 months with good tolerance (165).

### Nasopharyngeal carcinoma

The oncolytic adenovirus H101, with E1B-55KD and partial E3 deletion, was the first approved OV in China to treat head and neck cancer in 2005 (167). In a phase III randomized clinical trial, H101 was given intratumoral injection and combined with chemotherapy for treating squamous cell cancer of the head and neck or esophagus, and the ORR was 72.7% in the combination group versus 40.3% with chemotherapy alone. Injection site reactions were observed in 28.3% of patients, and 9.8% of patients had influenza-like symptoms (168, 169).

### Malignant glioma

Malignant glioma is aggressive, with a median OS of approximately 15 months (170). Glioma is restricted to the CNS, and local approaches, such as OVs, are an available therapy, including HSV1, adenoviruses, and polioviruses.

A single-arm phase II trial showed that a third-generation HSV1-based OV, Teserpaturev (G47Δ), demonstrated a 1-year OS of 84.2% in recurrent and/or residual glioblastoma patients with tolerable adverse events, such as fever, vomiting, nausea, and leukopenia and was approved in Japan for malignant glioma patients (2, 171). At the same time, G207, the oncolytic HSV1 strain, was assessed in pediatric glioma patients with high-grade glioma, and benefits were shown in nearly all patients (11/12) with a median OS of 12.2 months (172, 173) and only resulted in grade 1 adverse events.

Tasadenoturev, an adenovirus type 5-based OV, was assessed in 12 pediatric glioma patients with diffuse intrinsic pontine, and measurable shrinkage was observed in 75% of patients with a median OS of 17.8 months, but three events showed muscle weakness and headaches, grade 3 (174). Then, the tolerability and efficacy of tasadenoturev were evaluated in 37 adult recurrent glioma patients (174), and 20% of patients were still alive at 3 years after therapy, including 3 patients who had nearly complete responses (with ~95% tumor material loss) (175), with only 2 patients with treatment-related adverse events.

In addition to DNA viruses, RNA viruses, including poliovirus, are also being evaluated in clinical studies. PVS-RIPO, a recombinant attenuated poliovirus, was evaluated in a phase II study in patients with recurrent glioblastoma who received intratumoral PVS-RIPO and had a 1-year OS of 21% but with grade 3–5 adverse events in 19% of patients (including 1 treatment-related death) (176, 177).

### Bladder cancer

Nonmuscle invasive bladder cancer (NMIBC) is another indication for OV treatment, with most tumors arising in the urothelium of the bladder and are available for direct injections through intravesical infusions.

An adenovirus serotype 5-based OV, CG0070, encoding GM-CSF, was evaluated in a phase I/II trial and showed a complete response rate of 48.6% in a dose-dependent manner among 35 patients with a 10.4-month median duration of response (178). CG0070 demonstrated a 47% 6-month CR rate in another phase II trial in 45 patients with bacillus Calmette–Guerin (BCG)-refractory NMIBC with good toleration, and the most common therapy-related adverse reactions were bladder spasms, hematuria and dysuria (179). CG0070 in combination with ICIs, including pembrolizumab, was investigated with promising results (180) (NCT04387461).

### Pancreatic adenocarcinoma

Pancreatic adenocarcinoma is aggressive with a poor prognosis, with 1- and 5-year survival rates of ~18% and 7%, respectively. OVs are available therapies combined with traditional therapy in pancreatic tumors. Reovirus is a kind of ubiquitous double-stranded RNA virus to which most people have preexisting antibodies, but cancer cells with an activated RAS signaling pathway are more susceptible to reovirus (181). Reolysin, an oncolytic reovirus, has been assessed in clinical trials to evaluate its effect when combined with radiotherapy, immunotherapy, or chemotherapy (182, 183). In a phase Ib study with advanced-stage pancreatic adenocarcinoma patients, the combination of reolysin with immunotherapy (pembrolizumab) plus chemotherapy showed a 30% disease control rate (3 of the 10 evaluable patients), with 1 partial response lasting 17.4 months (184). In a phase II study in pancreatic adenocarcinoma patients receiving reolysin in combination with gemcitabine, the median OS was 10.2 months (182).

## Other solid tumors

There are studies on the use of OVs to treat other solid tumors, such as lung cancer and colorectal cancer. Seprehvir, an HSV1716-based OV, was assessed in a phase I/II trial with advanced-stage solid tumor patients involving children and young adults (11–30 years of age) and showed that two patients had stable disease in response to seprehvir (185). ONCR-177 was tested in phase I trials in advanced-stage and/or metastatic solid tumor patients as monotherapy or combined with pembrolizumab (186) (NCT04348916). Coxsackievirus-based OVs were evaluated alone and combined with ICIs in non-small-cell lung or bladder cancer patients (118).

## Conclusion and perspectives

OVs represent a new class of cancer treatments and hold great promise as novel cancer therapeutics. The main increasing interest in OV is the ability to induce lytic tumor cell death that promotes both innate and tumor-specific adaptive immune responses. At the same time, viruses are capable of being modified to enhance antitumor benefits by improving entry and replication in tumor cells and promoting antitumor immunity.

The clinical performance of OV monotherapy failed to match expectations to a certain extent, but combination therapy offered further promise with durable responses, especially combined ICI therapies. There is increasing evidence that the combinations of OVs with ICIs, or even ICIs encoded by OVs, may induce synergistic effects and have the potential to treat recalcitrant, immunologically cold tumors. The application of OV–ICI combinations is still in its early stage and is expected to develop along the traditional paradigm, not only for use in later lines for recurrent/metastatic disease but also for potential use in neoadjuvant and definitive chemoradiotherapy settings. However, the phase III clinical trial of MASTERKEY-265 (74) using OVs and ICIs has resulted in failure, suggesting the need for more exploration of combination therapy modalities, including dosing schemes, such as alternative, sequential or synchronous dosing, as well as dosing sequences and dosing interval patterns.

Like other therapeutic strategies, considerable preclinical and clinical challenges continue to impair OV development, such as incomplete understanding of the underlying mechanisms of tumor regression with specific OV agents, penetration into the tumor bulk, antiviral immune responses, off-target infection, adverse conditions in the tumor microenvironment, the lack of specific predictive and therapeutic biomarkers, and limited standardization of immune correlates in clinical trials. However, updated achievements to deepen the understanding of the mechanisms and immunology of OV therapy are guiding new OV tactics. With increased molecular understanding, a number of studies for optimizing oncolytic virotherapy are in clinical development.

The review of the clinical landscape of OV showed that the most common tumors targeted in OV cancer clinical trials were melanoma and gastrointestinal cancers, and the most common viruses used were adenovirus, HSV-1, reovirus and poxviruses (118), indicating that most clinical trials used DNA viruses with genetic modifications and transgene expression, such as GM-CSF. One meta-analysis by Li et al. reported that T-VEC showed remarkable efficacy with prolonged overall survival compared to other OVs, and the advantage of the objective response rate was identified with oncolytic DNA viruses via intratumoral injections but not in RNA viruses through intravenous injections (187). In general, solid tumors, especially those with sufficient infiltration of immune cells, are mainly targeted by oncolytic viruses.

Considering the many variables with OV therapies discussed, many directions are on the way for clinical trials, and the progress of treatment means will empower the full possibilities of OVs. There are many opportunities ahead for the development of these therapeutic strategies that may substantially improve cancer-related outcomes.

## Author contributions

LC: Writing – original draft, Writing – review & editing. MZ: Data curation, Investigation, Methodology, Writing – review & editing. QZ: Data curation, Investigation, Methodology, Writing – review & editing. YW: Conceptualization, Writing – review & editing.
